# Regulatory T Cells in Pregnancy: It Is Not All About FoxP3

**DOI:** 10.3389/fimmu.2020.01182

**Published:** 2020-06-23

**Authors:** Juliette Krop, Sebastiaan Heidt, Frans H. J. Claas, Michael Eikmans

**Affiliations:** Department of Immunohematology and Blood Transfusion, Leiden University Medical Center, Leiden, Netherlands

**Keywords:** regulatory T (Treg) cells, pregnancy, preeclampsia, Tr1 regulatory cells, Th3 regulatory cells, HLA-G Treg, immune tolerance, recurrent pregnancy loss (RPL)

## Abstract

In pregnancy, the semi-allogeneic fetus needs to be tolerated by the mother's immune system. Regulatory T cells (Tregs) play a prominent role in this process. Novel technologies allow for in-depth phenotyping of previously unidentified immune cell subsets, which has resulted in the appreciation of a vast heterogeneity of Treg subsets. Similar to other immunological events, there appears to be great diversity within the Treg population during pregnancy, both at the maternal-fetal interface as in the peripheral blood. Different Treg subsets have distinct phenotypes and various ways of functioning. Furthermore, the frequency of individual Treg subsets varies throughout gestation and is altered in aberrant pregnancies. This suggests that distinct Treg subsets play a role at different time points of gestation and that their role in maintaining healthy pregnancy is crucial, as reflected for instance by their reduced frequency in women with recurrent pregnancy loss. Since pregnancy is essential for the existence of mankind, multiple immune regulatory mechanisms and cell types are likely at play to assure successful pregnancy. Therefore, it is important to understand the complete microenvironment of the decidua, preferably in the context of the whole immune cell repertoire of the pregnant woman. So far, most studies have focused on a single mechanism or cell type, which often is the FoxP3 positive regulatory T cell when studying immune regulation. In this review, we instead focus on the contribution of FoxP3 negative Treg subsets to the decidual microenvironment and their possible role in pregnancy complications. Their phenotype, function, and effect in pregnancy are discussed.

## Placental Development and Immune Evasion by Trophoblasts

The most striking feature of pregnancy is that a semi-allogeneic fetus is tolerated by the maternal immune system. This is in sharp contrast with solid organ transplantation, where an allograft will be rejected by the patient's immune system unless the patient takes immunosuppressive drugs. Since direct contact between maternal and fetal cells occurs at the maternal-fetal interface in the placenta, it is thought that maternal immune cells in the placenta do not attack the fetal cells (trophoblasts) because of the tolerogenic microenvironment created by regulatory T cells (Tregs) and other immune cells.

### Trophoblast Development

**Figure 1:**
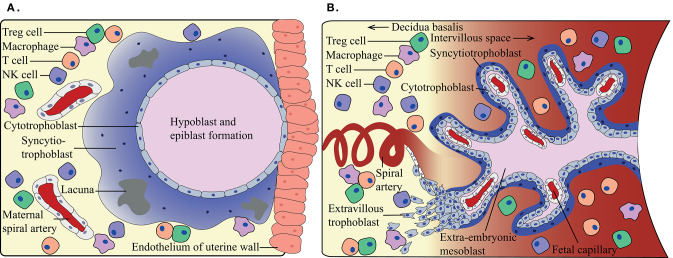
Schematic overview of the maternal-fetal interface at different trimesters. **(A)** During first-trimester, the maternal immune cells in the decidua can come into contact with fetal syncytiotrophoblasts, when around weeks 11–12 the maternal blood flow commences **(B)** a second maternal-fetal interface occurs. The maternal immune cells in the periphery can come into contact with fetal syncytiotrophoblasts, while the maternal decidual immune cells are in contact with the fetal extravillous trophoblasts. Indicating immunotolerance needs to adapt during the shift in gestation.

The main function of the placenta is to provide oxygen and nutrients to the developing fetus. In the first-trimester, nutrients are mainly provided by uterine glands in a hypoxic environment as no active maternal blood flow has been established yet. Once active maternal blood flow in the placenta has commenced around weeks 11–12 of gestation, oxygen and nutrients are exchanged over a thin lining of fetal cells. Since the fetus is semi-allogeneic, as it inherits both maternal and paternal antigens, the fetal trophoblast cells may potentially be recognized as foreign by maternal immune cells. Three main types of trophoblasts can be distinguished: cytotrophoblasts (CTBs), syncytiotrophoblasts (SCTs), and extravillous trophoblasts (EVTs). At the beginning of the first trimester, the maternal-fetal interface consists of the maternal parenchymal cells in the decidua and the fetal SCTs (Figure 1A). Later in pregnancy, this interface is mainly represented by maternal decidual cells and the EVTs (Figure 1B), where a distinction is made between decidua basalis and decidua parietalis. Importantly, a second maternal-fetal interface is established when active maternal blood flow in the placenta has commenced. The maternal peripheral blood then comes into contact with the SCTs lining the fetal villi. From the moment these maternal-fetal interfaces have been established, it is of utmost importance for maternal immune cells to keep the balance between tolerizing the semi-allogeneic fetus, and at the same time maintaining the ability to form a robust immune response against pathogens upon infection.

### Mechanisms by Trophoblasts for Avoiding and Modulating Immune Responses

The classical human leukocyte antigen (HLA) class I molecules HLA-A, -B, and -C are normally present on virtually all nucleated cells in the body and present intracellular antigens to surveilling T cells. Non-classical HLA molecules are selectively present, and have initially been described on trophoblasts in the placenta (1) and later also in other tissues (2–4). HLA class II is mainly expressed by antigen-presenting cells (APCs), including dendritic cells (DCs), macrophages, and B cells. Since the fetus inherits half of its genes from the father, it also inherits half of the paternal HLA alleles, which can potentially be recognized as foreign by the maternal immune system.

One way for the trophoblasts to evade recognition by the maternal immune system is lack of the polymorphic HLA-A, -B, and HLA class II molecules on their cell surface. Interestingly, EVTs do express polymorphic classical HLA-C molecules. The regular function of these molecules is to present a wide variety of pathogen-associated peptides to surveilling CD8^+^ T cells (5). Since HLA-C is polymorphic, its presence on trophoblasts can possibly also lead to allorecognition of the inherited paternal HLA-C by maternal T cells (6). EVTs may help to tip the local maternal immune balance toward tolerance by their expression of non-classical HLA-E and HLA-G (7), and possibly also HLA-F (8). The mechanisms responsible for the presence or absence of the specific HLA class I types on trophoblasts have not fully been elucidated yet (5). Expression of HLA molecules on trophoblasts allows them to escape natural killer (NK) cell recognition (9). HLA-G was first described on CTBs and has been shown to induce immune tolerance (10, 11) (described below). HLA-E also has tolerogenic properties as it can bind to the NK cell receptor CD94/NKG2A upon which NK cell activity is inhibited (12). SCTs, which are in direct contact with the maternal blood, do not express any HLA molecules (13), which would potentially render them sensitive to NK cell-mediated killing (13). However, for NK cells killing an activating ligand needs to be present on the target cell, which is likely missing on trophoblasts (14).

Trophoblasts express several molecules that are thought to dampen alloimmune reactivity, including PD-L1, PD-L2, CD200, and FasL (15–19), some of which are differentially expressed throughout gestation (17). Trophoblasts are also known to produce soluble factors with an immune-modulatory action, such as soluble HLA-G (sHLA-G), transforming growth factor-beta (TGF-β), and indoleamine 2,3-dioxygenase (IDO). TGF-β is known to have various functions and will be extensively discussed below. Since IDO causes local tryptophan deprivation (20), which is an essential amino acid required for T cell activation, elevated local IDO levels lead to inhibition of T cell activation. Recently, the role of galectins in pregnancy has become more apparent, as they were found to play an important role in suppressing the maternal immune system (21). Galectins on human trophoblasts modulate a number of regulatory mechanisms (22), such as induction of T cell apoptosis (23) and induction of Treg cell development (24).

### Maternal Immune Cells in the Decidua

Not only the composition of fetal cells in the placenta but also the composition of maternal immune cells changes throughout gestation. Already before conception, as early as seminal plasma exposure, activation and proliferation of fetus-specific maternal T cells in uterine draining lymph nodes have been observed in murine models (25). In humans, maternal APCs and CD8^+^ T cells seem to get recruited to the ectocervix upon coitus, but their specificity remains unknown (26). In the first trimester of human pregnancy, maternal leukocytes account for 30–40% of all cells in the decidua (27). During this period, the most prominent immune cells are decidual NK (dNK) cells (~60%), macrophages (~20%), and T cells (~10%) (27–29). During gestation, dNK cell frequencies decrease, macrophage frequencies remain relatively stable, and T cell frequencies increase (29). Next to these main immune cell populations, innate lymphoid cell (ILCs) other than NK cells, DCs, B cells, NKT cells, granulocytes, and mast cells are found in the decidua (30–32).

Despite the many mechanisms that trophoblasts have to evade an alloimmune response, fetus specific immune recognition has been observed in mice (33). Furthermore, fetus-specific CD8^+^ T cells (34, 35) and inherited paternal antigen (IPA)-specific antibodies are found in maternal peripheral blood during pregnancy (36–38). Both HLA-C and HLA-E restricted CD8^+^ T cells, specific for viral and bacterial peptides, are present in humans (39). However, maternal CD8^+^ T cells could recognize the paternally inherited HLA-C from the fetus or fetal minor histocompatibility antigens, and if not suppressed are likely to attack the fetal trophoblasts (34, 35). Besides this, ~30% of pregnancies result in the formation of paternal HLA-specific IgG antibodies (38, 40). Allo-antibodies directed against HLA-C of the fetus do not necessarily appear to be detrimental to pregnancy outcome (41), but some studies do show that they are associated with spontaneous preterm deliveries and recurrent pregnancy loss (RPL) (42, 43). Therefore, to inhibit the effect of maternal immune components, it is thought that local immune regulation is required to prevent anti-fetal immunity.

## Maternal TREG Cells During Gestation

To prevent a detrimental immune reaction against the fetus, maternal immune cells need to be regulated. The level of both FoxP3^+^ and Foxp3^−^ Tregs is increased in the peripheral blood of pregnant women compared to non-pregnant control women (44, 45). While the proportion of total T cells in the decidua is low during the first trimester (~10%), of which 10-30% of the CD4^+^ T cells are Tregs (28, 29, 46, 47), later in pregnancy the proportion of Tregs significantly increases in the decidua [(46); van der Zwan et al. submitted]. In mice the importance of Tregs during implantation and for maintenance of a healthy pregnancy is evident. This was shown in murine studies by injecting abortion prone mice with CD25^+^ Tregs from wild-type pregnant mice, which led to a significantly increased litter size (48). Alternatively, depleting CD25^+^ Tregs during the implantation period of non-synergistically mated mice caused high fetal resorption (49). Depleting Tregs in the mid-gestation phase in non-sterile mice also resulted in high fetal resorption (50). In a systematic review of 17 studies on human pregnancy, it has been shown that the number and functionality of Tregs are diminished in women experiencing RPL, both in the peripheral blood and in the decidua, compared to control women (51). Similarly, in women with pre-eclampsia decreased Treg frequencies in both the periphery and the decidua and impairment in the signaling of peripheral blood Tregs have been found (45, 52, 53).

Using extensive mass cytometry panels containing more than 38 immune cell markers, we have previously shown that there is great heterogeneity in immune cell subsets among the different trimesters (van der Zwan et al. submitted**)**. Interestingly, five Treg-like clusters were found to be differently distributed over the three trimesters. This could be attributed to the developmental changes in the placenta, causing a constant change in the possible cell-cell interactions between immune cells and different EVT subsets that seem to exist over different trimesters (54). Apart from that, a deficit in Treg presence and functionality has been observed in pregnancy complications such as PE, infertility, and RPL (55). Such complications arise at different periods of pregnancy, i.e., during implantation, <22–24 weeks of gestation or throughout gestation (56, 57). Taken together, as both Treg subsets and the initiation of complications can be prominent in a particular time frame of gestation, it might be that disbalances in different Treg subsets could play a role in the onset of different complications. Therefore, it is important to investigate the presence and functioning of the wide range of Treg subsets present during pregnancy.

## Advances in TREG Identification

Regulatory T cells were originally named suppressor cells (58). Ideas and insights changed over time, and suppressor cells have endured much debate. In 1983 it was shown in mice that both CD8 (Lyt-2^+^) and CD4 (Lyt-2^−^) suppressor cells were present that expressed the I-J molecule (59). When the I-J molecule turned out not to exist and suppressor cells could not be identified in any other way, interest in these cells waned. The arrival of novel molecular technologies propelled new knowledge, which made immunological tolerance become more evident and revived interest in T suppressor cells, now referred to as Tregs (60). In 2001, the *FoxP3* gene was identified in scurfy mice and later as a key transcription factor for Treg cell development and function in both humans and mice (61, 62). Subsequently, several FoxP3^−^ Treg subsets were identified, as will be discussed below. Initially, it was hypothesized that Tregs could only be generated in the thymus (tTregs), but in the 2000s this concept was challenged by studies showing that Tregs could be induced from conventional T cells in the periphery (pTregs) (63, 64). It is thought that tTregs and pTregs function in distinct ways, recognize different types of antigens (autoantigens vs. foreign antigens), and are needed in different immunological events such as preventing T cell trafficking to an organ and preventing T cell priming by APC, respectively (65).

Because tTregs and pTregs can have different roles, there is a need for phenotypic markers to distinguish the two. While Helios and Nrp-1 have been proposed as markers for tTregs in mice (66, 67), it has been shown that Helios deficiency or Nrp-1 deficiency does not impede tTreg development (65, 68). Consequently, there is no consensus on which markers can distinguish tTregs from pTregs (65, 69, 70). Helios is associated with the promoter regions of apoptosis/cell survival genes, and Helios deficient FoxP3^+^ Tregs show increased inflammatory cytokine expression, which suggests the importance of Helios in suppressing the production of effector cytokines (71). Even though Nrp-1 is not essential for tTreg development, it seems to increase Treg immunoregulatory properties, such as an increased capacity for tumor infiltration (69, 72). When comparing Nrp-1 and Helios there is no consistent overlap in expression of these markers (65). In humans, Helios is found on Tregs, but Nrp-1 is not found on peripheral blood Tregs and can, therefore, be excluded as tTreg marker (66, 73). More recently CNS1 has been suggested to distinguish between tTregs and pTregs. However, since CNS1 is a *FoxP3* enhancer, it is debatable whether this marker distinguishes FoxP3^−^ tTreg and pTreg populations (74, 75).

Treg subsets are often identified by their co-signaling molecules. Many Treg subsets express co-signaling molecules, such as ICOS, PD-1, TIGIT, and TIM-3, which upon interaction with their ligand can alter their function to either activation or senescence (76–78). These co-signaling molecules, which can be present on both FoxP3^+^ and FoxP3^−^ Tregs, have widely been discussed in several reviews (79–81). Similarly, the heterogeneity within FoxP3^+^ Tregs, generally described as CD4^+^CD25^+^CD127^−^ in functional assays, has been extensively reviewed elsewhere (82–87). However, the heterogeneity within the FoxP3^−^ compartment has not been elaborated on and will be discussed here in the context of pregnancy. Besides co-signaling molecules, several soluble factors affect the action of Tregs and are produced by these cells to mediate their immune regulatory effects. These will first be briefly reviewed.

## Soluble Factors

### IL-10

IL-10 is an immunomodulatory cytokine that is produced by many immune cells in the decidua, including most known Treg subsets. It has an effect on trophoblasts and innate- and adaptive immune cells within the decidua (88). Single nucleotide polymorphisms (SNPs) in the promoter region of IL-10 correlate with adverse pregnancy outcomes in humans (89). Next to that, the administration of recombinant IL-10 or IL-10 producing B cells to mice leads to reduced incidence of fetal resorption (90). Concomitantly, IL-10 null mice in sterile cages showed normal litter size, whereas administration of a danger signal in the form of a low dose of LPS to these mice resulted in increased fetal resorption (91, 92). These data suggest that IL-10 is an important mediator of immune regulation during pregnancy. In human pregnancy, decreased serum IL-10 levels or IL-10 production by PBMCs are associated with the occurrence of PE and RPL (93–98). This suggests that IL-10 producing immune cells are important for maintaining an uncomplicated pregnancy.

IL-10 induces expression of HLA-G on trophoblasts, which has direct and indirect immune suppressive effects (described below) (99). IL-10, together with HLA-G, can induce monocyte-derived DCs *in vitro* to differentiate into tolerogenic DCs (DC-10) that have immunosuppressive properties (100, 101). They exert their immunosuppressive properties by the production of IL-10, expression of HLA-G, and upregulation of inhibitory receptors for HLA-G (namely ILT2, ILT3, and ILT4). Furthermore, these tolerogenic DCs downregulate co-stimulatory molecules CD80 and CD86, as well as HLA-DR (102–104). DC-10s induce Tregs by their expression of ILT4 and by IL-10 production (105). Macrophages are also regulated by IL-10 (106). It has been shown that IL-10 acts on macrophages by controlling their metabolic pathways, causing activation, proliferation, and inflammatory responses to be inhibited (106, 107). Next to that, CD4^+^ T cell proliferation is suppressed by IL-10, antigen-experienced specific CD4^+^ T cells can be induced into an anergic state, and conventional T cells can be induced to convert to Tregs (103, 108–110).

### TGF-β

TGF-β is produced by and has an immunomodulatory effect on multiple cell types present in the decidua (111–120). In the early implantation phase, TGF-β is important for trophoblast invasion in the endometrium (121, 122). In humans, TGF-β serum levels are elevated in pregnant women compared to non-pregnant women, and serum levels are higher in early pregnancy compared to late pregnancy (123). However, women experiencing RPL display a decrease in TGF-β serum levels compared to women undergoing elective termination for non-medical reasons (124). Interestingly, there are indications from mouse studies that TGF-β induced Tregs could prevent spontaneous abortion, but this effect needs to be elucidated further (111, 125).

TGF-β can inhibit NK cell and T cell activation and proliferation by repressing the mammalian target of rapamycin (mTOR) signaling pathway (126, 127), and similarly, suppress activation of dNK cells (120). Since dNK cells are important contributors to angiogenesis at the maternal-fetal interface, their cytotoxicity needs to be suppressed but they should still be able to execute their role in angiogenesis. A balanced TGF-β level may, therefore, be important to maintain correct functioning of dNK cells (120). Furthermore, TGF-β can affect T cells directly by inhibiting their proliferation and differentiation (128, 129), and indirectly by its inhibitory effect on APCs. HLA-class II on APCs is downregulated, activation of macrophages is downregulated, and maturation of DCs is prevented by TGF-β (116, 130–134). Next to that, the presence of TGF-β is needed for the induction of several FoxP3^+^ and FoxP3^−^ Treg subsets by APCs (135–138).

### HLA-G

As discussed above, HLA-G was first described on trophoblasts (1). Interestingly, also myeloid and lymphoid cells, such as the below described FoxP3^−^HLA-G^+^ Treg, can express HLA-G and secrete sHLA-G (139–141). HLA-G is oligomorphic and has seven isoforms, of which some are membrane-bound (HLA-G1 to -G4), and others are secreted as a soluble form (sHLA-G5 to -G7) (142). Several polymorphisms in the untranslated region (UTR) of the *HLA-G* gene have been associated with lower sHLA-G levels in both blood and seminal plasma (143, 144). In both PE and RPL, a reduction in serum sHLA-G levels has been observed compared to healthy control women (145–148). Together these observations highlight the possible importance of (s)HLA-G during pregnancy.

(s)HLA-G exerts its immunoregulatory effects on a wide variety of immune cells because of its interactions with several inhibitory receptors, of which ILT2 seems to be most prominent (149). Other receptors for (s)HLA-G are ILT4, KIR2DL4, and CD8. The ITL2 receptor is expressed on monocytes/macrophages, DCs, B cells, and some NK and T cells (150), while the ILT4 receptor is mainly present on macrophages, NK cells, and neutrophils (150, 151). Upon ILT2 or ILT4 binding to HLA-G, NK cells and T cells receive a signal that leads to inhibited killing capacity (152–154). In CD8^+^ T cells, this inhibited killing capacity is reflected by the down-regulation of granzyme B expression (155). KIR2DL4 has been identified on dNK cells and some T cell subsets. Engagement of this receptor with sHLA-G results in activation and secretion of different types of cytokines and chemokines, but does not result in direct cytotoxicity (156). Binding of sHLA-G with KIR2DL4 on NK cells results in the upregulation of a restricted set of chemokines and cytokines that can promote vascular remodeling (156). CD8 is not only expressed by cytotoxic T cells but also by some NK cell subsets (79, 157). When sHLA-G binds to CD8, this interaction inhibits cytotoxic activity and triggers FasL-mediated apoptosis in both the CD8^+^ T cells and CD8^+^ NK cells (158). Besides effector cells, APCs can also be affected by HLA-G. For example, in concert with IL-10, HLA-G induces DCs to differentiate into tolerogenic DC-10 cells (100, 101). Additionally, macrophages obtain a tolerogenic phenotype upon binding to HLA-G with their ILT2 or ILT4, and subsequently show reduced expression of HLA class II, CD80, and CD86. Such macrophages have been described to be similar to decidual macrophages as they also express IDO (159). Together this suggests that decidual macrophages are under the constant influence of HLA-G, produced by either trophoblasts or HLA-G^+^ Tregs.

## Foxp3^−^ Regulatory T Cells

### FoxP3^−^ HLA-G^+^ Tregs

**Figure 2:**
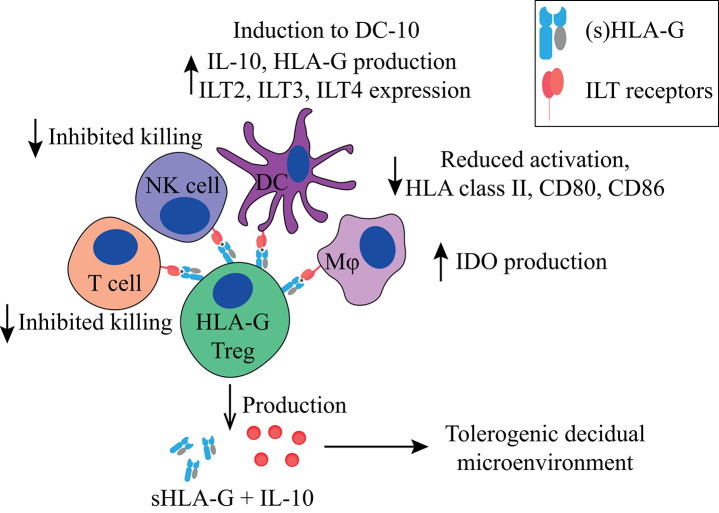
Main tolerogenic mechanisms of the FoxP3^−^HLA-G^+^ regulatory T cell. HLA-G^+^ regulatory T cells can suppress in a cell-contact dependent manner via HLA-G1. It inhibits the killing capacity of T cells and NK cells, downregulates HLA class II, CD80, and CD86 in DCs and macrophages, and makes them tolerogenic by inducing IDO production in macrophages and the induction of DCs to DC-10 cells. HLA-G^+^ regulatory T cells produce IL-10 and soluble HLA-G5 that helps to create a tolerogenic decidual microenvironment. HLA, human leukocyte antigen; NK, natural killer cell; DC, dendritic cell; IDO, indoleamine 2,3-dioxygenase; IL-10, interleukin-10; DC-10, tolerogenic DCs.

In the lymphoid compartment, HLA-G expressing CD4^+^ and CD8^+^ cells show reduced proliferation in response to allogeneic and polyclonal stimuli (139). CD4^+^HLA-G^+^CD25^−^FoxP3^−^ Tregs (Figures 2, 5, Table 1) suppress T cell proliferation through the expression of membrane-bound HLA-G1 and secretion of IL-10 and sHLA-G5 in a reversible, cell-contact independent and cell-contact dependent manner (139, 169). They have functionally been compared to other Treg populations such as FoxP3^+^ Tregs and Tr1 Tregs (discussed below), and represent a population that is distinct from tTregs (169–171). Interestingly, CD4^+^ and CD8^+^ T cells can also acquire a similar HLA-G1^+^ phenotype *in vitro* through trogocytosis (160), meaning the uptake of membrane fragments from another cell. Resting and activated CD25^+^ T cells that acquire HLA-G1 expression by trogocytosis differ functionally from the HLA-G^+^ tTregs, and they do not secrete sHLA-G5 and IL-10. They have been shown to exert their immune-suppressive capacity in a cell-contact dependent manner only (160), and will not be discussed further.

**Table 1:** Overview of FoxP3^−^ immune regulating T cells discussed in this review, how they are induced or activated, their main suppressive mechanism and how they function, their localization, animal models depletion assays, master genes for differentiation, and cell volume changes in complicated pregnancies.

**Subset**	**Induction/ activation**	**Suppressive mechanism**	**Function**	**Localization**	**Depletion in animal models**	**Master genes of differentiation**	**Cell volume changes in complications**
CD4^+^HLA-G^+^ Treg (139, 160)	Natural occurring (139)	Secretion of sHLA-G and IL-10 (139), and cell interaction with HLA-G (160)	Induction of HLA-G expression by trophoblasts, DC-10s and Tregs by IL-10 Inhibition of macrophages, NK cells and T cell killing	Found in peripheral blood (45) and decidua (141)	Has not been performed	Not known	Found to be increased in peripheral blood of pre-eclampsia patients (45)
Tr1-(like) Treg (109, 119)	Via trogocytosis (160)	Secretion of IL-10 and TGF-β, and cell interaction (136, 161)	Induction of HLA-G expression by trophoblasts, DC-10s and Tregs by IL-10 Lysis of APCs, disruption of metabolic state of T cells	Found in peripheral blood and decidua (119)	Has not been performed	Not known	Has not been described
Th3 Treg (162)	By APC in an IL-10 dominant microenvironment (110)	Secretion of TGF-β and IL-10 (162)	Induction of HLA-G expression by trophoblasts, DC-10s and Tregs by IL-10 Inhibition of NK cell and T cells and APC by TGF-β	Found in the decidua (163)	Has not been performed	Not known	Has not been described
CD8^+^ Treg (59)	By APC in presence of TGF-β and IL-4 (113, 135)	Suppress the secretion of immunoglobulins (164)	Prevent formation and suppressing production of IPA-specific antibodies.	Found in peripheral blood (CD8^+^HLA-G^+^ Treg) (45) and decidua (164)	Has not been performed	Not known	CD8^+^HLA-G^+^ Treg are increased in peripheral blood of pre-eclampsia patients (45)
NO-Treg (165)	CD101^+^CD103^+^ are induced by trophoblasts (164)	Secretion of IL-10 (165, 166)	Induction of HLA-G expression by trophoblasts, DC-10s and Tregs by IL-10.	Found in peripheral blood (165)	Has not been performed	Not known	Has not been described
TIGIT^+^ Treg (119)	Depends on nitric oxide, p53, IL-2, and OX-40 (165)	Secretion of IFNγ and IL-2 (119)	Induction of IL-10 production by APCs. Suppression of CD4^+^ effector T cells	Found in decidua (119)	Has not been performed	Not known	Has not been described
Vδ1^+^ γδ T cell (167)	Unknown	Secretion of IL-10 and TGF-β (115)	Induction of HLA-G expression by trophoblasts, DC-10s and Tregs by IL-10 Inhibition of NK cell and T cells and APC by TGF-β	Found in peripheral blood and decidua (168)	Has not been performed	Not known	Decreased amount in an abortion prone mice model (111)

HLA-G^+^ tTregs accumulate at sites of inflammation to regulate immune responses (172) and importantly, have also been found in the decidua (141, 173). CD4^+^HLA-G^+^ Treg frequencies are increased in peripheral blood throughout pregnancy compared to non-pregnant controls (45, 141). Interestingly, sHLA-G serum levels are also increased during pregnancy, while these levels are decreased in complicated pregnancies compared to healthy pregnancies (145–148). However, it is unlikely that a direct correlation between CD4^+^HLA-G^+^ Treg frequencies and serum sHLA-G levels exists, since other cells (in the placenta) produce sHLA-G as well. CD4^+^HLA-G^+^ Treg frequencies within the CD4^+^ T cell compartment are even higher in the decidua compared to those in peripheral blood (141, 173), suggesting a role in local immune regulation. In women with PE, decidual CD4^+^HLA-G^+^ Tregs are decreased, whereas in the peripheral blood their numbers remain unchanged compared to healthy control pregnancies (45, 173), indicating that in a healthy pregnancy these cells are induced locally, but to a lesser extent during PE.

### Tr1 Treg

**Figure 3:**
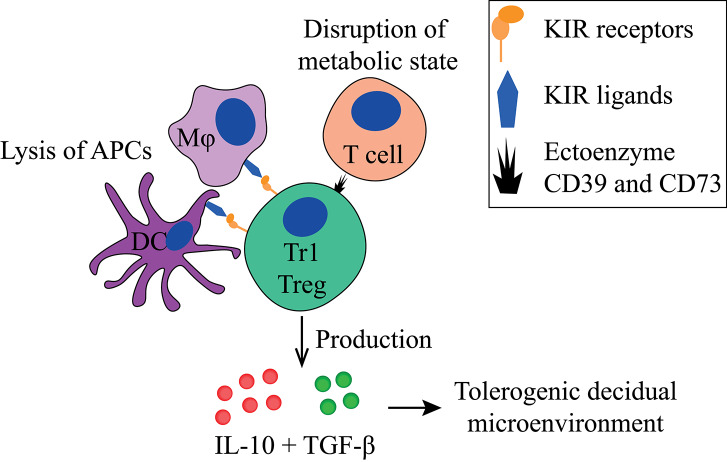
Main tolerogenic mechanisms of the Tr1 regulatory T cell. Tr1 regulatory T cells can in a cell-contact dependent manner lyse APCs via their KIR receptors and disturb the metabolic state of T cells. They produce IL-10 and TGF-β that helps to create a tolerogenic decidual microenvironment. APC, antigen-presenting cell; KIR, killer-cell immunoglobulin-like receptor; IL-10, interleukin-10; TGF-β, transforming growth factor-beta.

Tr1 Tregs (Figures 3, 5, Table 1) suppresses T cell proliferation mainly through IL-10 and TGF-β production. They also produce low amounts of IFN-γ, IL-5, and IL-2, and express granzyme B (109, 112, 174). Next to cytokine production, they can suppress other immune cells in a cell-contact dependent manner by using their KIR receptors or ectoenzymes (161). Tr1 Tregs are peripherally induced upon chronic antigen stimulation in the presence of IL-10 (175). Both HLA-G and IL-10 provided by APCs, like DC-10 cells, play a role in Tr1 Treg induction (103), indicated by their reduced induction by DC-10s when anti-HLA-G is added *in vitro*. Additionally, their induction is reverted when agonistic anti-ILT4 antibodies are added, but not when agonistic anti-ILT2 antibodies are added (103). Interestingly, EVTs are also able to induce Tr1-like cells via HLA-G directly (119).

Recently, co-expression of CD49b and LAG-3 has been described as phenotypic markers for Tr1 Tregs in mice and humans (176). This observation is under debate since a subsequent study only detected a small proportion of IL-10^+^ Tregs co-expressing CD49b and LAG3 (177). Due to their lack of a clear phenotype, Tr1 Tregs are often described as Tr1-like cells, as they have similar properties, such as IL-10 production. Tr1 Tregs can express the co-signaling molecules PD-1, CTLA-4, TIM-3, and ICOS (136, 177–179), and several other molecules related to their function, including GARP, LAP, ectoenzyme CD39, and CD73 (180), as well as KIRs and ILT receptors. FoxP3 is only transiently expressed by Tr1 Tregs. Since functional Tr1 Tregs are found in patients who have a mutation in the *FoxP3* gene, FoxP3 appears not to be required for their development (110, 174).

Tr1-like Tregs have been identified in peripheral blood and various tissues (181), including the human decidua (119). These Tregs express high levels of PD-1, express granzymes, and lack FoxP3. They produce IL-10 and IFN-γ, and thereby may have a similar suppressive mechanism as bona fide Tr1 Tregs (119). Similar to Tr1 Treg, decidual Tr1-like Treg induction by EVTs can be partially reverted when agonistic anti-HLA-G antibodies are added, but not by anti-ILT2 (119). Tr1 Tregs are able to selectively lyse APCs in a cell-contact dependent manner, but not B and T cells (161). Lysis of APCs can cause amplification of the tolerogenic process since decreased numbers of activated APCs will generally lead to less activation of T cells. For this, the Tr1 Treg needs HLA-class I recognition of the APC through its KIR receptors, CD54/LFA-1 mediated adhesion, CD58/CD2 interaction, as well as CD155/CD226 ligation (161). Furthermore, the Tr1 has been described to directly affect T cells by their expression of ectoenzyme CD39 and CD73, which disrupts the metabolic state of effector T cells (180).

### Th3 Tregs

**Figure 4:**
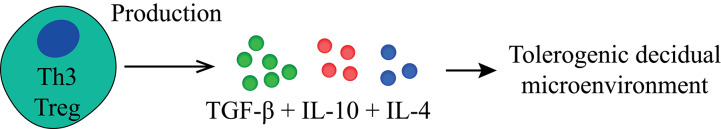
Main tolerogenic mechanisms of the Th3 regulatory T cell. Th3 regulatory T cells suppress in a cell-contact independent mechanism only by the production of TGF-β, IL-10 and differ here from the Tr1 regulatory T cell by the production of IL-4. TGF-β, transforming growth factor-beta; IL, interleukin.

**Figure 5:**
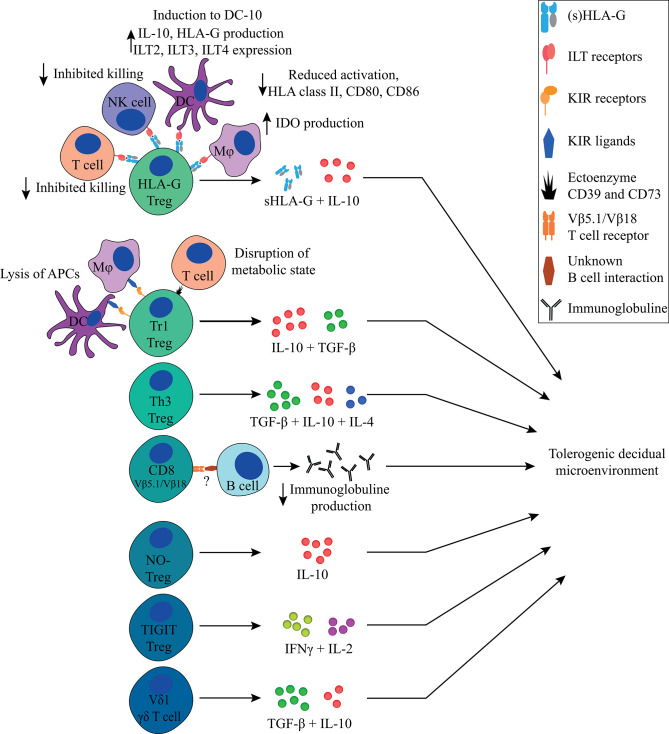
Overview of FoxP3^−^ immune regulating T cells discussed in this review and their main tolerogenic mechanisms in pregnancy. All Tregs described in this review can exert immunosuppressive properties in a cell-contact independent mechanism that together contributes to a tolerogenic decidual microenvironment. Next to that, the HLA-G^+^ Treg, Tr1 Treg, and CD8^+^ Treg can exert their immunosuppressive properties in a cell-contact dependent mechanism.

The main suppressive effects of Th3 Tregs (Figures 4, 5, Table 1) are mediated by TGF-β production, in a cell-contact independent manner (135). Phenotypically these cells are CD25^−^ and FoxP3^−^, they are thought to express Helios, and express LAP and GARP, which can be used as surrogate markers for TGF-β production (182, 183). Th3 cells also produce IL-10, but unlike Tr1 Tregs, they produce this in conjunction with IL-4 (113, 184). Similar to Tr1 Tregs, Th3 Tregs are peripherally induced upon antigen stimulation (135). The mechanism underlying the induction into either Th3- or Tr1 Treg remains poorly understood and is thought to depend on their microenvironment during priming (114, 185). Another question that remains to be answered is whether Tr1 and Th3 Tregs truly represent different subsets or differentiation states and whether they differ depending on the microenvironment in which they reside.

With the limited markers identified so far, it is difficult to phenotypically identify Th3 Tregs, which may explain the limited number of articles describing the presence of the Th3 cell during pregnancy. Dimova et al. observed in paired decidua and peripheral blood samples mRNA cytokine profiles similar to Th3, the first description of a possible presence of Th3 cells in the decidua (163). Importantly, no functional testing has been performed for Th3-like cells from the decidua, and their presence and role in pregnancy remains to be confirmed. Regardless, Th3 Treg was first described to have an important role in oral tolerance (182). Interestingly, exposure to semen through oral sex has been proposed to be beneficial for subsequent pregnancy outcomes in couples experiencing PE or RPL (186–188), providing a possible mechanistic explanation for this effect.

### Other Treg Populations

Besides FoxP3^−^ HLA-G^+^, Tr1, and Th3 Tregs, other immune regulatory T cell populations that have been described, albeit to a lesser extent, include CD8^+^ Tregs, nitric oxide (NO) induced FoxP3^−^ Tregs, TIGIT^+^ Tregs, FoxP3^dim^ Tregs, and γδ T cells (Figure 5, Table 1).

CD8^+^ Tregs are increasingly being recognized, even though they remain difficult to identify as there is no consensus on their phenotype. Both FoxP3^+^ and FoxP3^−^ CD8^+^ Tregs have been described to have suppressive activities, indicating there also is heterogeneity in the CD8^+^ Treg population (189). Shao et al. showed that a CD8^+^ Treg subset can be activated by trophoblast cells. This activation appears not to be HLA restricted since their expansion is unaffected when cultured in the presence of pan-HLA class I blocking antibodies (164). When cultured with PBMCs, these CD8^+^ Tregs suppress the secretion of immunoglobulins in a cell-contact dependent manner, as shown using a trans-well system. While humoral immunity seemed to be dampened, these CD8^+^ Tregs did not have any suppressive effect on effector T cells. Phenotypically these cells can be identified as being CD101^+^ and CD103^+^ (164). Even though in a mixed lymphocyte reaction these CD8+ Tregs do not appear to suppress CD4^+^ and CD8^+^ T cells, they could potentially be important for preventing formation and suppressing production of IPA-specific antibodies.

Niedbala et al. described NO-induced Tregs (NO-Tregs) in mice (165). These cells are characterized as CD4^+^CD25^+^GITR^+^CD27^+^T-bet^low^, GATA3^+^, and FoxP3^−^, and they are induced from CD4^+^CD25^−^ T cells via p53, IL-2, and OX-40 (165). Experimentally, the development of NO-Tregs was induced when using adoptive transfer of CD4^+^CD25^−^ T cells into SCID mice, together with application of an NO synthase inhibitor. NO-Tregs produce IL-4 and IL-10, but no IL-2, TGF-β, or IFN-γ. Addition of antagonistic anti-IL4 antibodies led to reduced proliferation of NO-Tregs, whereas blocking IL-10 blocked their suppressive effect on CD4^+^CD25^−^ cell differentiation (165, 166). These data suggest that NO-Tregs suppress through IL-10, in a cell-contact independent manner. While NO-Tregs has not yet been studied in the context of pregnancy, NO appears to be involved in pregnancy with NO levels fluctuating throughout the different gestational ages and being lower during PE (190–193). It would, therefore, be interesting to retrospectively study first-trimester blood samples of women who develop PE, to test if NO levels are already lower at this early time point of pregnancy, and to study NO-Treg formation in these patients in comparison to healthy controls.

Salvany-Celades et al. identified three types of functional Tregs in the decidua, of which two subsets were negative or low for FoxP3 (119). One of these is the PD-1^high^, Tr1-like cell, which has been described above. The second is the TIGIT^+^ Treg that is characterized by TIGIT positivity, low expression of CD25 and FoxP3, and intermediate expression of PD-1. TIGIT^+^ Tregs express high levels of IFN-γ and IL-2, and low levels of IL-10. TIGIT^+^ Tregs mainly suppress CD4^+^ effector T cells in proliferation assays, but not consistently CD8^+^ effector T cells. Interestingly, TIGIT^+^ Tregs seem to vary in their characteristics, depending on the trimester in which they are encountered (119): first-trimester TIGIT^+^ Tregs show an increased expression of IL-10 compared to term TIGIT^+^ Tregs. This difference in trimesters could be due to the microenvironment influencing their phenotype, or because they truly represent different subsets. TIGIT has been described to be expressed on multiple Treg subsets, and it can bind CD155 on APCs, which thereby increases their IL-10 production (194, 195). Binding of TIGIT induces Tregs to produce IL-10 and fibrinogen-like protein 2 (Fgl2). By usage of Fgl2 the Tregs obtain the capacity to suppress Th1 and Th17 cells *in vitro*, but not Th2 cells (77, 195). It would be interesting to determine the presence of TIGIT^+^ Tregs during pregnancy complications and to investigate their possible role in providing a tolerogenic microenvironment in successful pregnancies.

In the first-trimester decidua, γδ T cells produce high amounts of IL-10 and TGF-β (115, 196). As described above, these cytokines are important for establishing an immune suppressive microenvironment in the decidua. Transfer of uterine γδ T cell culture supernatant, containing a high concentration of TGF-β, into the uterus of mice before pregnancy prevents fetal resorption (111). Terzieva et al. identified the TCR repertoire from decidual γδ T cells and compared this to the repertoire of γδ T cells in peripheral blood. In 1^st^ and 3^rd^ trimester decidua they mostly found Vδ1^+^ TCR, whereas this particular δ chain was hardly present in the peripheral blood (168). Vδ1^+^ T cells are described to have a tolerogenic effect (167, 197). The possible role of γδ T cells in pregnancy is further suggested by another study showing higher numbers of γδ T cells in peripheral blood from women experiencing RPL compared to controls. The specific presence of the Vδ1 chain was not investigated (198). It would be interesting to determine the frequency and immune-suppressive effect of Vδ1^+^ T cells in the decidua of women experiencing RPL compared to women with elective termination of pregnancy.

## Concluding Remarks

In this review we have discussed several types of Tregs that may contribute to a tolerogenic environment in the decidua (Figure 5, Table 1) besides FoxP3^+^ Tregs. Decidual Tregs seem to assist other cells in creating and maintaining a microenvironment where inflammatory signals are generally overruled by tolerogenic signals. Next to Tregs, this tolerogenic microenvironment is established and maintained by factors from paternal, maternal and fetal origin. Paternal contribution to this tolerogenic microenvironment comes early on from seminal fluid that contains tolerogenic factors such as TGF-β and paternal antigens for priming. Fetal trophoblasts contribute by their expression of tolerogenic HLA-G and HLA-E molecules, galectins, and PD-L1, and by their production of sHLA-G, IDO, and TGF-β. Next to this, the maternal contribution in maintaining a tolerogenic microenvironment in the decidua is provided by the decidual immune cells, which do not have an activated phenotype and produce IDO, TGF-β, IL-10, and sHLA-G.

It remains to be elucidated which mechanisms exactly attract Tregs to the decidua, if they are activated locally by APCs in the decidua or in the lymph nodes, where they proliferate, and if they are specific for fetal antigens. In mice, it has been shown that fetus-specific Tregs are already detectable in the uterine draining lymph nodes shortly after semen exposure and that their numbers increase upon pregnancy (199). While this could be similar in the human situation, *in vitro* fertilization with donor semen, where there is no paternal semen exposure, often results in a healthy uncomplicated pregnancy, albeit at a lower rate than in naturally conceived pregnancies (200). More information on the basic mechanisms of FoxP3^−^ Tregs, as well as how they are initiated, is needed to provide insight in the deviations in frequencies or functionality of FoxP3^−^ Treg subsets in pregnancy complications. Likewise, from a therapeutic point of view such basic mechanisms need to be clarified before possible novel therapeutic strategies can be developed. These therapies could be based on therapy designs similar to those proposed for FoxP3^+^ Tregs, such as infusion of Tregs or application of the cytokines needed for induction of specific Treg subsets (201).

While it is clear that FoxP3^+^ Tregs play a role in maintaining pregnancy, the relevance of the different types of FoxP3^−^ Tregs herein needs to be established. FoxP3^−^ Tregs with proven suppressive capacities are found in the decidua and are, therefore, likely to contribute to the tolerogenic microenvironment. However, studies such as depletion assays in mice need to be performed to confirm whether they play a non-redundant role in maintaining a healthy pregnancy. Since pregnancy is crucial for the existence of mankind, it is not surprising that there would be multiple mechanisms in play to establish a regulatory microenvironment to maintain a healthy pregnancy. Pregnancy complications for which no clear cause can be identified do occur, and it is plausible that many of these are related to a disbalance in maternal immune regulation. It would be helpful to get a better understanding of the function of all regulatory T cells present in the decidua, to be able to recognize their relevance in healthy and complicated pregnancies. As such, the use of multiple omics techniques to identify the decidual microenvironment by a holistic approach could give insights in the presence, frequency, and distribution of the different types of Tregs in pregnancy [(32, 202, 203); van der Zwan et al., submitted]. It is important to note that the time point of sampling is a crucial factor in such experiments, given the dynamic nature of the placental microenvironment.

## Author Contributions

JK contributed to the content design, writing the manuscript, and preparing the figures. FC, SH, and ME supervised the project and participated in critical discussions and evaluations of the text of the manuscript.

## Conflict of Interest

The authors declare that the research was conducted in the absence of any commercial or financial relationships that could be construed as a potential conflict of interest.

## Abbreviations

Tregs: regulatory T cells
tTreg: thymic derived regulatory T cell
pTreg: periphery induced regulatory T cell
EVTs: cytotrophoblasts (CTBs), syncytiotrophoblasts (SCTs) and extravillous trophoblasts
HLA: human leukocyte antigen
APCs: antigen presenting cells
KIR: killer-cell immunoglobulin-like receptor
TCR: T cell receptor
IDO: indoleamine 2,3-dioxygenase
dNK: decidual NK
RPL: recurrent pregnancy loss
PE: pre-eclampsia
SNPs: single nucleotide polymorphisms
NK: natural killer
ILCs: innate lymphoid cell
DCs: dendritic cells
DC-10: tolerogenic DCs
mTOR: mammalian target of rapamycin
NO: nitric oxide
TGF-β: transforming growth factor-beta
IFN-γ: interferon gamma.
